# HIF1α-dependent and independent pathways regulate the expression of PD-L1 in prostate cancer

**DOI:** 10.1007/s12032-023-02017-6

**Published:** 2023-04-17

**Authors:** Erasmia T. Xanthopoulou, Christos Kakouratos, Christos Nanos, Anastasia G. Gkegka, Christos Kalaitzis, Alexandra Giatromanolaki, Michael I. Koukourakis

**Affiliations:** 1 Department of Radiotherapy/Oncology, Democritus University of Thrace, 68100 Alexandroupolis, Greece; 2 Department of Pathology, Democritus University of Thrace, 68100 Alexandroupolis, Greece; 3 Department of Urology, Democritus University of Thrace, 68100 Alexandroupolis, Greece

**Keywords:** Prostate cancer, PD-L1, Hypoxia, HIF1α, IFNγ, Radiotherapy

## Abstract

PD-L1/PD-1 pathway is a major pathway exploited by human cancer types, which is a target for current immunotherapy. We investigated tumor microenvironmental factors involved in PD-L1 induction in prostate cancer (PC). We studied the expression of PD-L1 in a series of 66 PCs, in parallel with the expression of hypoxia- and acidity-related immunohistochemical markers (Hypoxia-inducible factor HIF1α, and lactate dehydrogenase LDHA) and tumor-infiltrating lymphocyte TIL density. Experiments with three PC cell lines, the 22Rv1, DU145, and PC3 were conducted focusing on the inducibility of PD-L1 by hypoxia, acidity, lymphocyte interactions, and radiation. In tissues, PD-L1 expression by cancer cells was directly related to PD-L1 expression by  TILs and macrophages (*p* < 0.05), and the overexpression of HIF1α and LDH5 (*p* < 0.05). TIL density was inversely related to ΗΙF1α (*p* = 0.02). Exposure of PC cell lines to hypoxia strongly induced PD-L1 and protein and mRNA levels, directly controlled by HIF1α function (*p* < 0.001). Irradiation with 20 Gy had no apparent effect on PD-L1 expression. Culturing PC cell lines with culture medium (CM) from PBMCs strongly induced PD-L1 at protein and mRNA levels, independently from HIF1α, which was also confirmed when cells were incubated with Interferon-γ (*p* < 0.001). It is concluded that the combination of anti-PD-L1/PD-1 immunotherapy with hypoxia/HIF-targeting may be important in the treatment of specific subgroups of PC patients.

## Introduction

Blocking of immune-control checkpoint molecular pathways (ICMPs) has emerged as a promising strategy for cancer therapy. Recent studies have prompted a lot of understanding of the immunotherapy-related mechanisms targeting ICMPs [1]. The binding of the PD-1 (programmed cell death protein 1) T-cell (T-lymphocytes) receptor to the PD-L1 (programmed death ligand 1) ligand expressed by regulatory immune cells or cancer cells restricts normal T-cell function. The PD-1/PD-L1 mechanism is essential for normal immune physiology, as it suppresses immune over-activation allowing tolerance to various antigens and preventing autoimmune reactions [2]. By exploiting this very immunosuppressive pathway, cancer cells suppress the antitumor immune response [3].

Over the years, studies have also brought forward the critical role of the tumor microenvironment (TME) in the repression of the cytotoxic function of tumor-infiltrating immune cells. Poor tumor vascularization and impaired blood flow due to an immature vascular network produce a hypoxic TME. Cancer cells undergo a reprogramming of their metabolic profile characterized by intense glycolysis and anaerobic usage of pyruvate, even under aerobic environmental conditions [4]. Glycolytic metabolism involves lactate dehydrogenase A (LDHA) overexpression so that pyruvate is converted to lactic acid, one of the principal causes of intratumoral acidity [5]. Several recent studies suggest that a hypoxic and acidic tumor microenvironment prevents lymphocyte proliferation and the antitumor activity of T-cells and natural killer NK-cells, also promoting the accumulation of myeloid and M2-type macrophages with immunosuppressive activity [6–10]. Moreover, PD-L1 gene has been postulated to be under the direct control of the Hypoxia-Inducible Factor 1α (HIF1α) [11].

Tumor-infiltrating lymphocytes and monocytes secrete immunostimulatory or immunosuppressive cytokines, including interferons (IFNs), interleukins (ILs), tumor necrosis factors (TNFs), or transforming growth factor-β (TGFβ). The biological activity of such cytokines on cancer cells' expression of immune checkpoint molecules (ICMs) is under intense investigation. Up-regulation of the PD-L1 expression in cancer cells by such cytokines has been reported [12]. IFN-γ, for example, induces PD-L1 expression in a dose-dependent manner, which raises concerns, as cytokine-secreting antitumor immune cells may produce a cancer cell defensive response that blocks immunity [13, 14].

In the current study, we investigated the role of intratumoral hypoxia and anaerobic metabolism in the expression of PD-L1 in prostate cancer (PC) at the tissue level and in vitro experiments. We provide direct evidence that HIF1α and IFNγ are involved in distinct molecular pathways controlling the up-regulation of PD-L1 expression in this common human malignancy.

## Materials and methods

### Tissue immunohistochemistry

The immunohistochemical method used to detect PD-L1, HIF1α, and LDH5 in formalin-fixed paraffin-embedded PC tissues has been reported in previous studies of ours [15, 16]**.** PD-L1 protein was detected with the rabbit monoclonal anti-PD-L1 antibody [clone CAL10; BiocareMedical, CA, USA, dilution 1:1000, 60 min incubation]. For the detection of LDH5 (composed of 4 LDHA subunits), we used the Ab9002 (Abcam, UK) sheep polyclonal antibody raised against the human placenta LDH5 from at dilution of 1:200, and overnight incubation. The HIF1α protein was detected using the mouse monoclonal ESEE122 antibody (gift by professor K.C. Gatter, Oxford, UK), raised against a recombinant fragment corresponding to human HIF1 alpha aa 300–550 (dilution 1:200, overnight incubation).

For PD-L1, the percentage of cancer cells with strong membrane (with or without cytoplasmic) expression was recorded in the entire tissue section in × 200 optical fields, and the mean value was used to score each case. Membrane staining in at least 1% of cancer cells was considered for PD-L1 positivity. The extent of PD-L1 expression in tumor-infiltrating lymphocytes and monocytes (TILMs) in the tumor stroma was assessed in all available optical fields. The percentage of stroma area with PD-L1 + TILMs was used to score cases. Moreover, the tumor-infiltrating lymphocyte TIL density was also assessed in hematoxylin and eosin sections by counting the number of TILs per optical field in all available optical fields covering the tumor and using the mean value to score each case. For HIF1α and LDH5 immunostaining, the proportion of tumor cells expressing cytoplasmic and/or nuclear reactivity was recorded after examining the entire tumor area at × 200 magnification. The median value was used to score each case. Expression in ≥ 50% of cells defined cases with high HIF1α or LDH5 expression.

### Cell lines and cultures

PC cell line 22Rv1 was purchased from the American Type Culture Collection (https:// www.lgcstandards-atcc.org/Products/All/CRL-2505.aspx, Manassas, USA), and the PC cell lines DU145 and PC3 were purchased from the CLS Germany (http://clsgmbh.de/p1699_PC-3.html and http://clsgmbh.de/p708_DU-145.html). The cell lines were cultured under aseptic conditions using DMEM Low Glucose (Dulbecco's Modified Eagle Medium) medium [Material: LM-D1102/500; Batch no: MS008W; ID no: MS008W100R; Biosera, UK], supplemented with 10% (v/v) Fetal Bovine Serum [Cat no: FB-1001/500; Biosera, UK] and 1% (v/v) Penicillin/ Streptomycin buffer [Cat no: LM-A4118/100; Biosera, UK], was used to properly grow the cell cultures. The validation of the Authentication of the PC cell lines was issued by Eurofins-Forensik, Germany. Cell cultures are maintained in the Incubator (Heraeus, 12L01I001) at a constant temperature of 37° C, 95% humidity, and 5% CO2.

### Hypoxia and acidosis conditions

For the hypoxia conditions, PC cell lines 22Rv1, DU145, and PC3 were seeded in 6-well plates, in an approximate yield of 2 × 105 cells per well. After 24 h, cells were incubated in hypoxic conditions in FLUOstar® Omega (BMGLABTECH) for 24 h in mixed gas airflow, consisting of 5% CO2, 1% O2, and 74% N2. For the acidosis conditions, PC cell lines 22Rv1, DU145, and PC3 were seeded in 6-well plates, in an approximate yield of 2 × 105 cells per well. After 24 h, cells were incubated in culture medium DMEM (Dulbecco's Modified Eagle Medium) containing 18.12 mmol/L HCl [37%; CL00.0310.2500; CHEM-LABS] (pH = 6.5) for 24 h. After that, protein isolation for Western Blot Analysis and RNA isolation protocol for Real-Time PCR (Polymerase Chain Reaction) experiments was performed, following the experimental procedures mentioned below.

### RNA interference procedure

Prostate cancer cell lines 22Rv1, DU145, and PC3 were stably transfected with a second-generation Lentiviral system via two plasmid vectors purchased from GenePharma [Shanghai GenePharma, China], the first one encoding unique sequences for the shRNA (Small hairpin RNAs) of HIF1α gene [LV10N(U6/mCherry&Puro); HIF1A-homo-964; Sequence: 5′GCTGATTTGTGAACCCATTCC3′] and the other one free of any coding sequence. Both plasmids included a single restriction site for the EcoRI, genes conferring resistance in Ampicillin and Puromycin, and a gene expressing the mCherry protein. Plasmid amplification was performed on DH5-Alpha competent bacteria cells. The selection procedure of the successfully transformed bacterial clones was performed via Ampicillin. The amplified-plasmid DNA extraction and purification were performed using the NucleoSpin Plasmid Miniprep kit (REF#740,588.10, Macherey–Nagel, Germany). Transient transfection of the host cell line HEK293T with the lentiviral vector [LV10N (U6/mCherry&Puro&Amp)] was performed in order to produce the lentiviral particles. The lentiviral vector was combined with the adequate plasmids, an envelope plasmid [pczVSV-G; cat#8454, Addgene] and a packaging plasmid [pCMVR8.74; cat#22,036, Addgene]. The aforementioned transfection was performed using Lipofectamine2000 transfection reagent [cat#11,668,019, Thermo-Fisher Scientifc Inc., USA]. Lentiviral particles, produced from HEK293T cells, were isolated from the supernatant, centrifuged at 1000 g for 10 min, filtrated and used to infect 22Rv1, DU145, and PC3 cells for 48 h. Successfully transfected cells were selected using Puromycin at a concentration of 1 μg/mL to 4 μg/mL.

### Co-Cultures and irradiation protocol

PC cell lines 22Rv1, DU145, and PC3, cell lines containing a non-coding (nc) plasmid, and cell lines with permanent silencing of HIF1α gene, were exposed to 20 Gy of irradiation, using the 6-MV beam produced by an I Infinity™ linear accelerator (Elekta, Stockholm, Sweden). The medium of these cultures was used to incubate the respective cancer cell lines for two days (matched types). Moreover, cell lines were incubated with culture medium obtained from PBMCs from a healthy donor cultured for 2 days in DMEM cell culture medium. PD-L1 expression was evaluated after 48 h via western blot analysis.

### Reagents and chemicals

#### Chrysin

Parental PC cell lines DU145 and PC3 with high basal levels of PD-L1 gene and protein were incubated for 24 h with 50 μmol/L of Chrysin [Chrysin; Lot. no: L27075/A; Enzo Life Sciences].

#### Interferon-gamma

PC cell lines 22Rv1, DU145, and PC3, cell lines containing a non-coding plasmid, and cell lines with permanent silencing of the HIF1α gene were incubated with 25 ng/ml of IFNγ for 48 h. IFNγ was purchased from PeproTech [Recombinant Human IFN-γ; Lot. no: 091927; Cat. no: 300-02; PeproTech, USA].

### Western blot analysis

Cell lysates were collected using RIPA lysis buffer [RIPA Buffer; Lot. No: #SLCD5849; Sigma-Aldrich, USA], enriched with the appropriate volume of Protease Inhibitor Cocktail [10x, lyophilized powder; Lot. No: #2896346; Cat. No: #20-201; EMD Millipore Corporation, Temecula, CA, USA], as well as phosphate Phosphate Inhibitor Cocktail Set V [50x; Lot. No: #3029143; Cat. No: #524629; EMD Millipore Corporation, Temecula, CA, USA] and then homogenized using a plastic pestle. Total protein concentration was calculated using the BCA (Bicinchoninic acid) Protein Assay Thermo Scientific ™ Pierce ™ [Pierce™ BCA Protein Assay kit; Lot. no: #PG206436; Thermo Scientific, USA]. The Western Blot analysis was performed using Primary Rabbit Monoclonal Anti-PD-L1 [1:200; Lot. no: 080317; Cat. no: ACI3171A,C; BIOCARE MEDICAL, CA, USA], Sheep Polyclonal Anti-lactate Dehydrogenase Isoenzyme 5 antibody [1:1000; #ab9002; Abcam, UK], as well as Purified Mouse anti-Human Hif1a antibody [Lot. no: 9049717; Cat. no: 610959; 150 μg, 0.6 ml, 250 μg/ml; BD Transduction Laboratories, USA]. Furthermore, 30 μg for PD-L1 and LDH5, and 18 μg for HIF1a protein were loaded for analysis. Protein samples were separated on discontinuous SDS gels using 15%, 10%, and 8% separating gels for PD-L1, LDH5, and HIF1a proteins, respectively, and 6% stacking gel. Electroblotting was performed with PVDF membrane [Immobilon-P PVDF for WB analysis, 26.6 × 1.8, pore size 0.45um; Cat. no IPVH00005; Merck Millipore, Germany], and then membranes were blocked with 5% Non-fat dry milk in 150 mM NaCl, pH 7.5 10 mM Tris (TBS), and 0.1% (v/v) Tween 20 at 20–25 °C for 1 h followed by overnight hybridization with primary antibodies at 4 °C. The membranes were then hybridized for 2 h at room temperature with the secondary antibodies Goat Anti-rabbit IgG (H + L)-HRP Conjugated [1:1000; #1706515; BioRad, UK], Polyclonal Rabbit Anti-Sheep Immunoglobulins/HRP [1:1000; #0062180; DAKO, Denmark], and Goat Anti-mouse IgG (H + L)-HRP Conjugated [1:1000; #1706516; BioRad, UK], for PD-L1, LDH5, and HIF1a proteins, respectively. Finally, membranes were developed using the ChemiDoc MP Imaging System [Bio-Rad Laboratories, CA, USA]. Each blot was then stripped, dried overnight, re-hybridized with Mouse Monoclonal Anti-beta-actin (HRP) antibody [1:20000; ab49900; abcam, UK], and Rabbit Monoclonal Anti-Lamin-B1 [1:1000; #ab133741; Abcam, UK]. All experiments were performed three times, and the densitometric analysis of proteins was performed using the Image Lab software [Bio-Rad Laboratories, CA, USA].

### Real-time PCR (RT-PCR)

The RNA isolation protocol NucleoSpin® RNA Plus [Lot. No: 1807/002; REF: 740,984.50; Macherey–Nagel GmbH Co. & KG, Düren, Germany] kit was used for the isolation of RNA, following the steps provided by the kit. Total RNA was measured using NanoDrop 200° C (Thermo Fisher Scientific, Waltham, Massachusetts, USA), and the PrimerScript RT Reagent Kit (#RR037A; Takara, Shiga, Japan) was used to construct the cDNA. The expression levels of particular genes were measured using real-time quantitative PCR and the SensiMix SYBR No-ROX KitThe [KAPA SYBR FAST qPCR kit; # KK4611; KAPA Biosystems Inc., Roche, Basel, Switzerland] optimized for LightCycler® 480 [KK4611, KapaBiosystems, USA]. 20 µl of final reaction volume was prepared containing 10 ng of cDNA and master mix at final concentrations: 1X KAPA SYBR FAST qPCR Master Mix (2X): 200 nM forward primer: 200 nM reverse primer and ddH2O. Reaction cycles were carried out in 4 steps: activation of the enzyme for one cycle at 95 °C for 3 min, amplification for 40 cycles at 95 °C for 10 s, 56 °C for 20 s and 72 °C for 1 s, melting curves recovered. 95 °C for 5 s and 65 °C for 1 min and cool to 40 °C for 10 s. For each sample, 3 measurements of the transcriptional activity of the gene of interest were taken, and normalization was based on a gene with the same transcriptional activity in each sample. The relative changes between the transcriptional levels of the genes of interest were calculated using the comparative Ct method (2 − ΔΔCt) as the efficiencies between the target genes and reference gene are approximately equal and near to 100%. The design of the primers for LDHA (primer sequence 5′→3’ forward/reverse: GCAGATTTGGCAGAGAGTATAATG/GACATCATCCTTTATTCCGTAAAGA), PD-L1 (primer sequence 5′→3’ forward/ reverse: AAATGGAACCTGGCGAAAGC/GATGAGCCCCTCAGGCATTT), HIF1a (primer sequence 5′→3’ forward/reverse: TTTTTCAAGCAGTAGGAATTGGA/GTGATGTAGTAGCTGCATGATCG) and HPRT (primer sequence 5′→3’ forward/ reverse: GATTAGCGATGATGAACCAGGTT/CCTCCCATCTCCTTCATGACA), genes was performed with the Roche primer design tool (https://lifescience.roche.com/en_gr/brands/universal-probe-library.html#assaydesign-center), the Eurofins Genomics primer design tool [Eurofins Genomics, North America], and the TIB Molbiol Genomics Sequencing Primer Planning Tool [TIB Molbiol Genomics, Syntheselabor GmbH Eresburgstr, Berlin], respectively, and the results were quantified with LightCycler software (LightCycler® 480 SW 1.5.0 SP4 v1.5.0.39, Roche, Switzerland).

### Statistical analysis

Graph presentation and statistical analysis were performed by using the GraphPad Prism 8 version. The paired or unpaired two-tailed t test was applied for analysis of groups of continuous variables, as appropriate. A *p* value of < 0.05 was considered for significance.

## Results

### PD-L1 expression in PC tissues

**Fig. 1:**
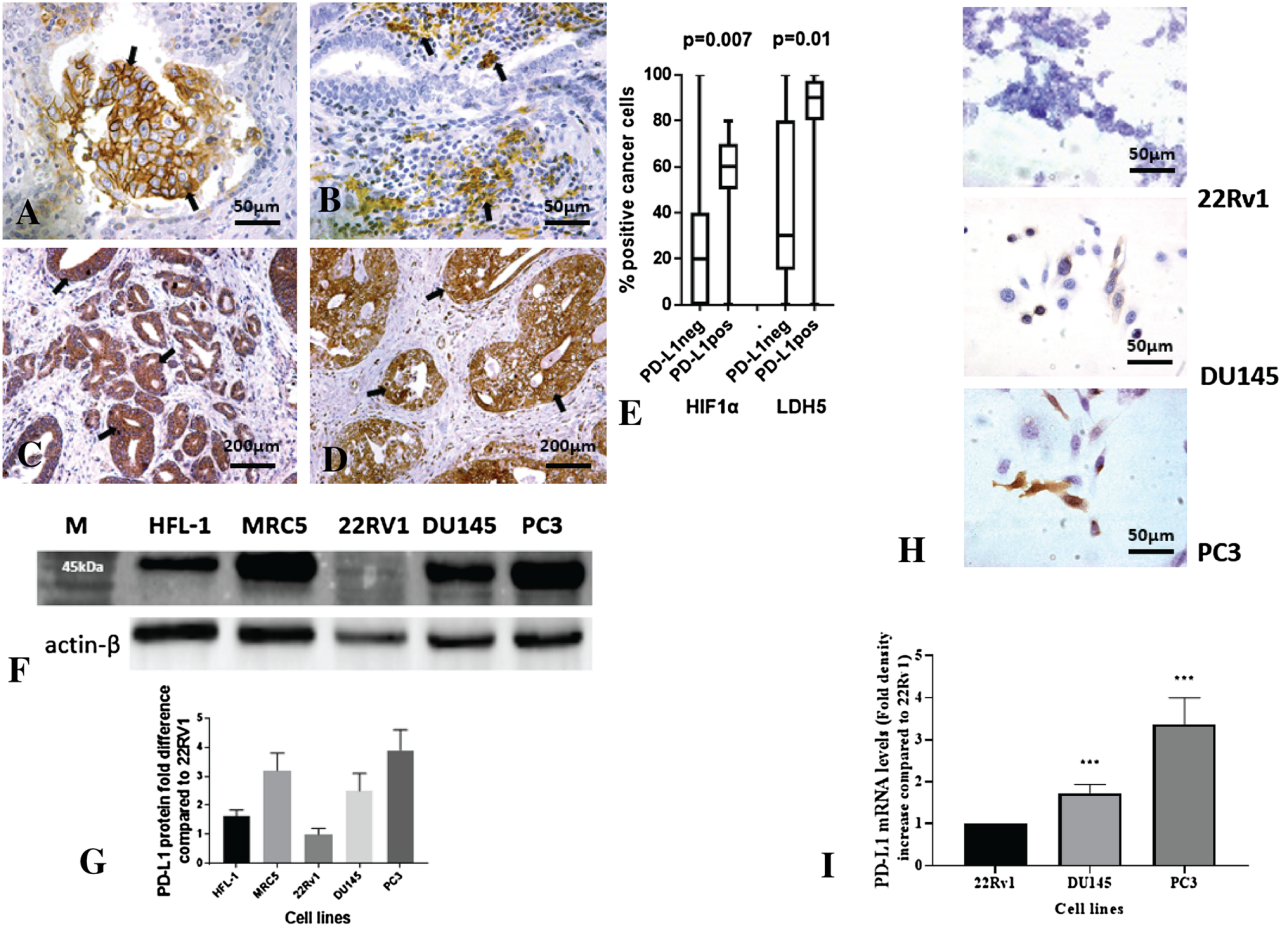
Immunohistochemical analysis of PC tissues: **A** Typical membrane expression of PD-L1 in cancer cells (arrows; magnification × 40); **B** Expression of PD-L1 in stroma infiltrating lymphocytes and monocytes (arrows; magnification × 40); **C** A PC tumor with nuclear and cytoplasmic expression of HIF1α (arrows; magnification × 20); **D** A PC tumor with nuclear and cytoplasmic expression of LDH5 (arrows; magnification × 20); **E** The % of cancer cells expressing HIF1α and LDH5 according to the expression of PD-L1 (negative vs. positive). Box and whiskers graphs showing the median value, range, and 25th/75th percentiles. (**F,G,H,I**). Basal levels of PD-L1 expression: **F** western blot images showing the basal expression levels of PD-L1 protein in PC cell lines 22Rv1, DU145, and PC3 in parallel with two human fibroblast cell lines (HFL1 and MRC5); **G** band densitometry of the western blot (p values are referred to the comparison between 22Rv1 and the other two PC cell lines); **H** typical immunocytochemical expression of PD-L1 expression levels in PC cell lines 22Rv1, DU145, PC3 (magnification × 40); **I** RT-PCR analysis of the mRNA expression of the PD-L1 gene, in PC cell lines DU145, PC3, in comparison with the expression in 22Rv1. (***p < 0.001). (Abbreviations: M = marker, *** = *p* < 0.001)

Fifty-six PC tissues from patients who underwent prostatectomy were examined. Membrane (with or without cytoplasmic) PD-L1 expression in > 1% of cancer cells was noted in 13 of the cases (23.2%; positive cases). The percentage of cancer cells expressing PD-L1 in these cases ranged from 1 to 60% (median 5%). 8/56 (14.3%) cases expressed PD-L1 in 1–9% and 5/56 (8.9%) in 10–60% of cancer cells; Fig. 1A**.** Lymphocytes and macrophages infiltrating the tumor stroma (TILMs) expressed PD-L1 in 17/56 (30.3%) samples (positive samples). In 8/56 (14.3%) cases, PD-L1 + TILMs were noted in < 10% of the examined optical fields, and in 9/56 (16.1%) PD-L1 covered > 10% of optical fields; Fig. 1B. Expression of PD-L1 in cancer cells was directly related to PD-L1 expression in TILMs (*p* < 0.05). There was no association between PD-L1 expression (cancer cell or TILM) with Gleason score or T-stage.

Cytoplasmic/nuclear expression of HIF1α was evident in 36/56 (64.3%) cases and ranged from 0–100% of total cancer cells (median 30%); Fig. 1C. High expression (> 50% of cancer cells) was noted in 21/56 PCs. Cytoplasmic/nuclear expression of LDH5 was recorded in 45/56 (80.3%) cases and ranged from 0 to 100% of total cancer cells (median 70%); Fig. 1D. High LDH5 expression (> 50% positive cancer cells) was noted in 32/56 PCs.

PD-L1 expression by cancer cells was directly related to high HIF1α and high LDH5 expression in cancer cells; Fig. 1E**; **Table 1. The median percentage of HIF1α + cancer cells was 60% in cases with high PD-L1 cancer cell expression vs. 20% in cases with low (*p* < 0.01). The median percentage of LDH5 + cancer cells was 90% in tumors with high PD-L1 cancer cell expression vs. 30% in tumors with low (*p* < 0.05). Expression of PD-L1 by TILMs was not related to HIF1α or LDH5 expression.

**Table 1:** Immunohistochemical expression patterns of PD-L1, HIF1α, and LDH5

% positive cancer cells			
	PD-L1	HIF1α	LDH5
All cases (56 cases)			
Min	0	0	0
Max	60	100	100
Median	0	30	70
Mean	3.6	33.5	53.9
33rd percentile	0	0	20
67th percentile	0	50	82
PD-L1 negative case (43 cases)			
Min	0	0	0
Max	0	100	100
Median	0	20	30
Mean	0	27.4	46.8
33rd percentile	0	0	20
67th percentile	0	34	72
PD-L1 positive (13 cases)			
Min	1	0	0
Max	60	80	100
Median	5	69	90
Mean	15.3	53.8	77.3
33rd percentile	3.6	50	80
67th percentile	14.5	64	95

The tumor-infiltrating lymphocyte TIL density ranged from 6 to 520 lymphocytes per × 200 optical field. The median value was 96, and this was used to split PCs in two groups of low and high TIL density. Out of 21 PCs with high HIF1α expression, 6 (28.5%) had high TIL density, while out of 45 cases with low HIF1α expression, 22 (48.9%) had high TIL density (*p* < 0.05). There was no association between LDH5 expression and TIL density.

### Basal levels of PD-L1 in PC cell lines

Western blot analysis of PD-L1 expression in 22Rv1, DU145, and PC3 PC cell lines showed very low expression in the 22Rv1 hormone-sensitive cell line. In contrast, a robust expression was evident in the DU145 (2.6-fold compared to 22Rv1) and, especially, in the PC3 cell line (3.9-fold compared to 22Rv1) (Fig. 1F,G). The Western blot patterns of PD-L1 expression were also confirmed in immunocytochemistry, where the 22Rv1 was negative, while the DU145 and PC3 cell lines expressed PD-L1 in 20% and 30% of cancer cells, respectively (Fig. 1H). In RT-PCR, the DU14 and PC3 cell lines had 1.6- and 2.5-fold increased PD-L1 mRNA expression compared to 22Rv1 (Fig. 1I) (*p < 0.05, **p < 0.01, ***p < 0.001).

### Effects of hypoxia/acidity on PD-L1 expression

**Fig. 2:**
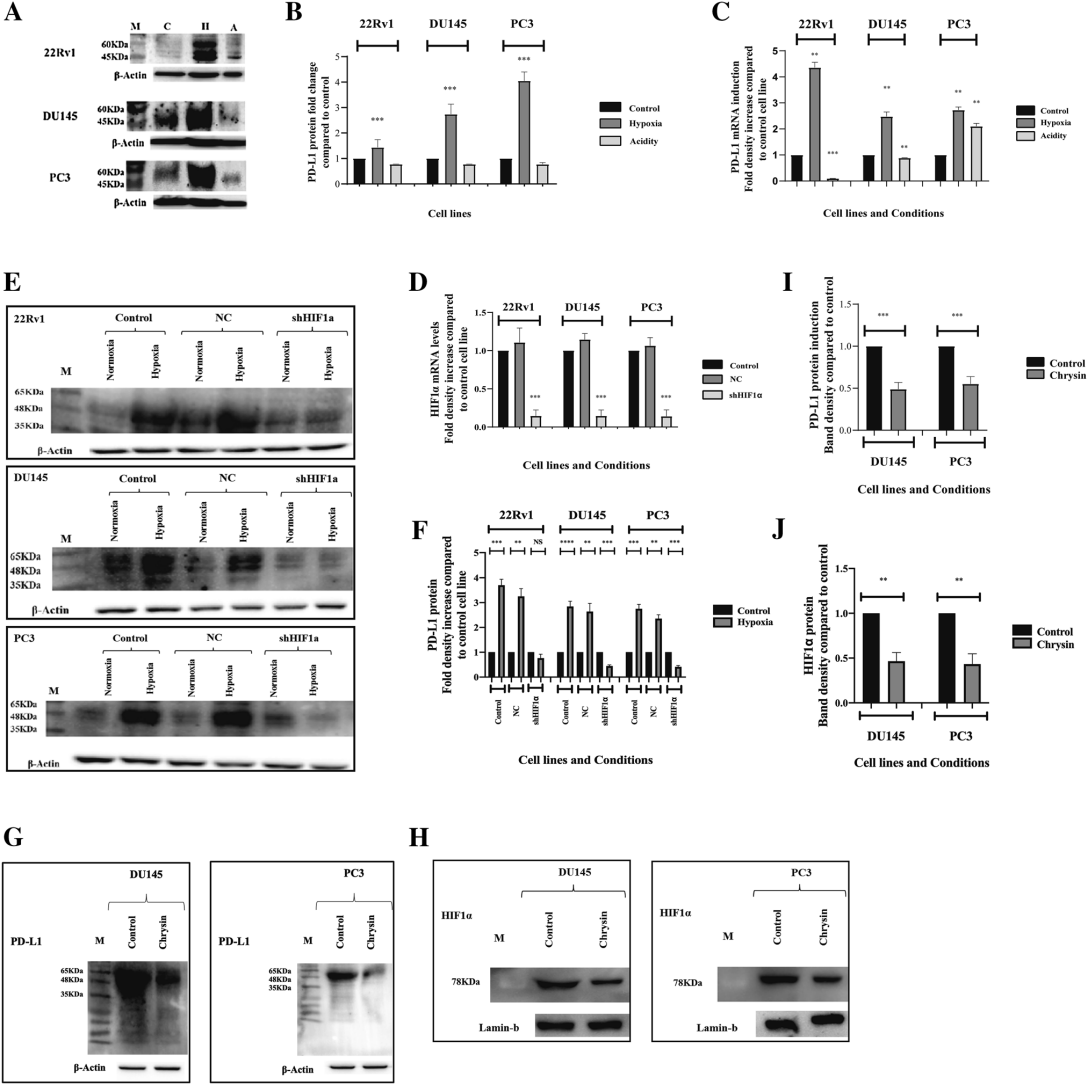
PD-L1 expression under hypoxic, acidic conditions and exposure to the HIF1α-inhibitor chrysin: **A & B** Western blot images and densitometry analysis of the expression of protein PD-L1. **C** RT-PCR analysis showing the PD-L1 mRNAs changes in the 22Rv1, DU145, and PC3 PC cell lines exposed to hypoxia and acidity. **D** RT-PCR analysis of the mRNA expression of the HIF1α gene under normoxic conditions of the 22Rv1, DU145, PC3 shHIF1α cell lines in comparison with the control cell lines and cell lines transfected with a non-coding sequence. **E** PD-L1 expression in western blot analysis of control PC cell lines, cell lines transfected with a non-coding sequence, and PC cell lines with permanent silencing of HI1α gene, and **F** band densitometry after exposure to hypoxia*.* **G** Western blot images of the effect of 50 μmol/L of chrysin on HIF1α protein levels in DU145 and PC3 prostate cancer cell lines and band densitometry. **H** Western blot images of the effect of 50 μmol/L of Chrysin on PD-L1 protein levels in DU145 and PC3 prostate cancer cell lines and band densitometry (*p < 0.05, **p < 0.01, ***p < 0.001). (Abbreviations: M = marker, NC = non-coding, shHIF1α = silenced HIF1α with small hairpin RNA, NS = not significant, * = *p* < 0.05, ** = *p* < 0.01, *** = *p* < 0.001)

PC cell lines were exposed to hypoxic and acidic conditions, as described in the methods. In western blot analysis, hypoxia strongly induced the expression of PD-L1 in all three cell lines (*p* < 0.001; Fig. 2A,B). This was also confirmed in RT-PCR (*p* < 0.01) (Fig. 2C). Acidity had a differential effect in PD-L1 mRNA expression among cell lines (suppressed in the 22Rv1, stable in the DU145, and increased in the PC3; Fig. 2C).

### Silenced HIF1α gene and response to hypoxia

The creation of stably transfected sh22Rv1, shDU145, shPC3 cell lines with shRNA of the HIF1α was confirmed with RT-PCR analysis (Fig. 2D). Gene expression of HIF1α was significantly reduced in all three PC cell lines (in 22Rv1 cell line 3.3-fold compared to control cell line, *p* < 0.001; in DU145 cell line 4.76-fold compared to control cell line, *p* < 0.001; in PC3 cell line 7.14-fold compared to control cell line, *p* < 0.001). Transfection with non-coding sequence (nc22Rv1, ncDU145, ncPC3) had no effect on HIF1α expression.

In western blot, exposure to hypoxia resulted in PD-L1 up-regulation in all three control cell lines and nc-cell lines (22Rv1 2.75-fold, *p* < 0.001; nc22Rv1 2,34-fold *p* < 0.01; DU145 2.84-fold *p* < 0.001; ncDU145 2.63-fold *p* < 0.01; PC3 3.69-fold, *p* < 0.001; ncPC3 cell line 3.25-fold *p* < 0.001). shHIF1α cell lines exhibited decreased or stable PD-L1 protein levels (sh22Rv1 1.1-fold change *p* = NS; shDU145 2.24-fold decrease *p* < 0.001; shPC3 2.43-fold decrease *p* < 0.001) (Fig. 2E,F).

### Effect of chrysin on HIF-1α expression

Exposure of PC cell lines DU145 and PC3 to 5 μmol/L chrysin repressed the expression levels of HIF1α protein (DU145 1.96-fold decrease *p* < 0.01; PC3 2.08-fold decrease *p* < 0.01) (Fig. 2G). The expression levels of PD-L1 protein were also decreased in both PC cell lines (DU145 2.32-fold decrease *p* < 0.001; PC3 twofold decrease *p* < 0.001) (Fig. 2H).

### Effect of irradiation and PBMC culture medium

**Fig. 3:**
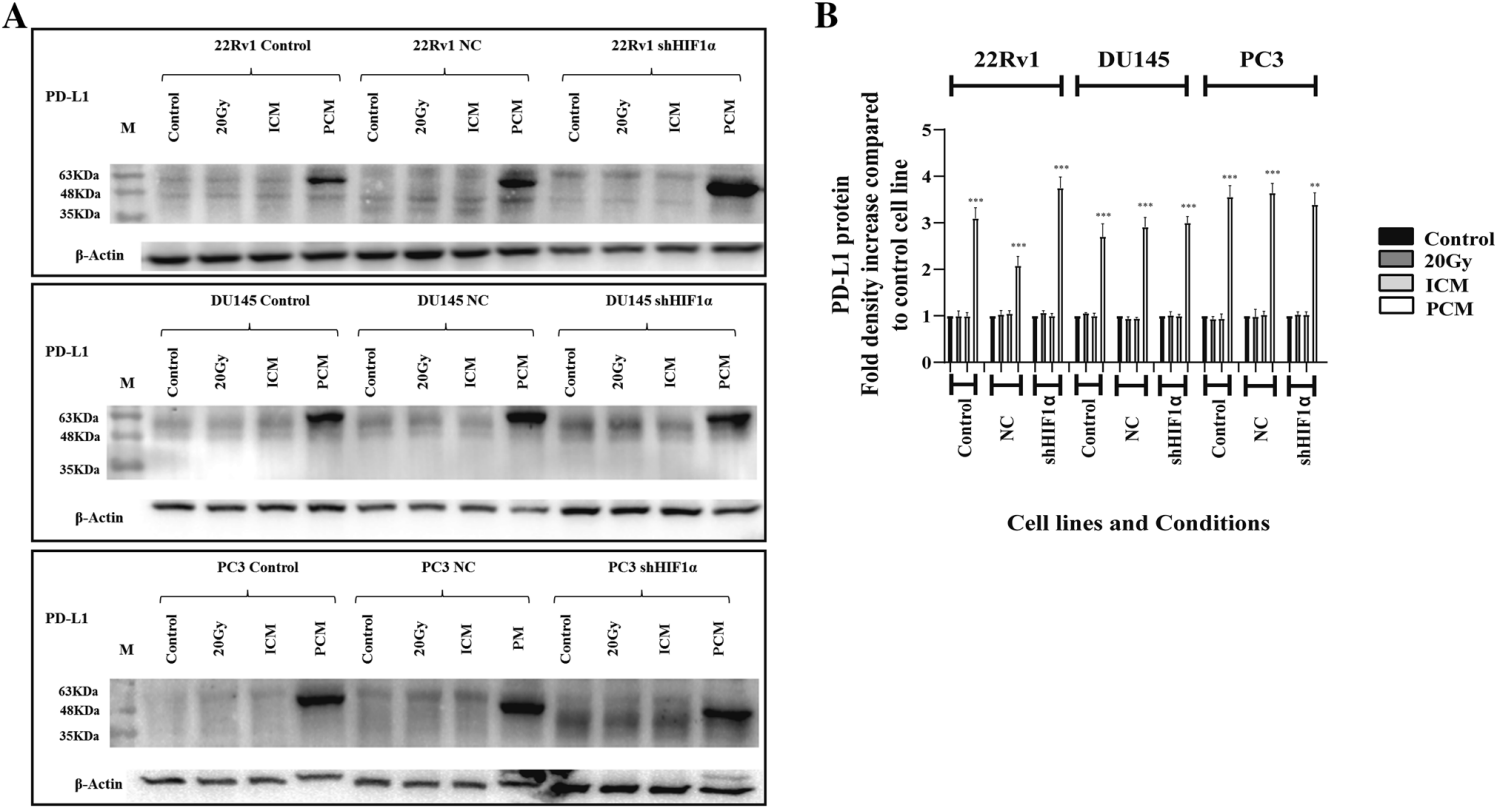
PD-L1 expression and response to direct 20 Gy cancer cell irradiation, exposure to culture medium from 20 Gy irradiated cancer cells (ICM; 2-day culture after irradiation) and culture medium from untreated PBMCs (PCM, 2-day culture): Western blot images **A** and band densitometry **B** showing the expression pattern of PD-L1 of the 22Rv1, DU145, PC3 (control, transfected with non-coding NC sequence or with shHIF1α) cell lines (*p < 0.05, **p < 0.01, ***p < 0.001).). (Abbreviations: M = marker, NC = non-coding, shHIF1α = silenced HIF1α with small hairpin RNA, ICM = culture medium form irradiated cancer cells, PCM = culture medium from untreated PBMCs, * = *p* < 0.05, ** = *p* < 0.01, *** = *p* < 0.001)

All three control, nc-cell lines and shHIF1a cell lines were i. irradiated with 20 Gy, ii. cultured in medium obtained from respective cancer cell lines two days after irradiation with 20 Gy, and iii. cultured in medium from PBMC cultured for 2 days in DMEM. Two days later, western blot analysis was performed to detect the levels of PD-L1 protein **(**Fig. 3A,B**)**. Irradiation and incubation of cancer cell lines with medium from pre-irradiated cancer cells did not affect PD-L1 levels. Culture medium from PBMCs, however, strongly induced the expression of PD-L1 in all cell lines (22Rv1 2.98-fold, *p* < 0.001; nc22Rv1 1.95-fold, *p* < 0.001; sh22Rv1 3.84-fold, *p* < 0.001; DU145 2.29-fold, *p* < 0.001; ncDU145 threefold, *p* < 0.001; shDU145 2.84-fold *p* < 0.001; PC3 3.4-fold, *p* < 0.001; ncPC3 3.83-fold, *p* < 0.001; shPC3 3.28-fold *p* < 0.01).

### Effect of IFNγ on PD-L1 expression

**Fig. 4:**
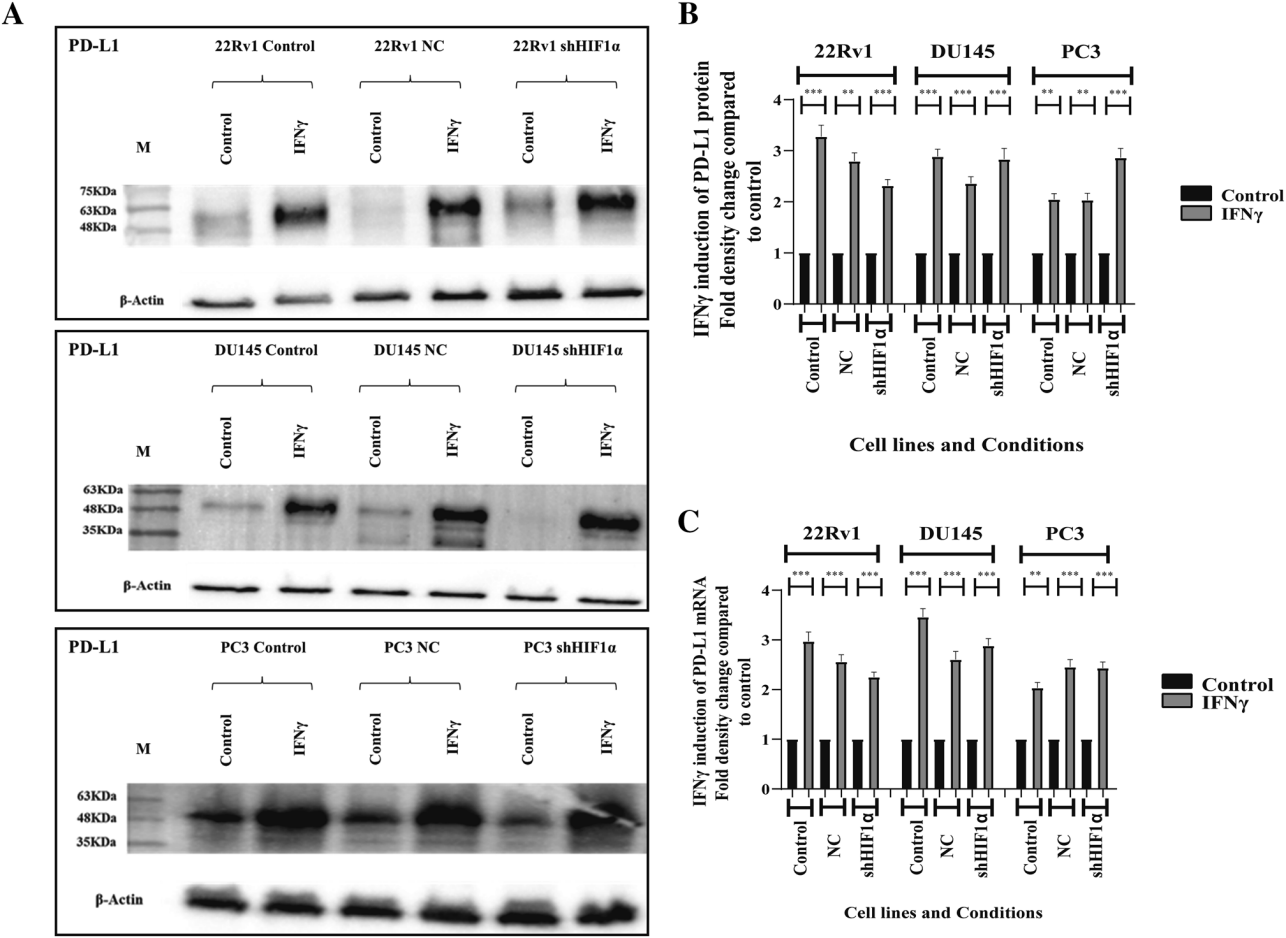
Effect of IFNγ on PD-L1 expression: **A** western blot images and **B** band densitometry showing the expression pattern of PD-L1 protein in control, nc- and sh- 22Rv1, DU145, PC3 cell lines after exposure to 25 ng/ml of IFNγ for 48 h. **C** RT-PCR analysis of the mRNA expression of the PD-L1 gene (*p < 0.05, **p < 0.01, ***p < 0.001). (Abbreviations: M = marker, NC = non-coding, shHIF1α = silenced HIF1α with small hairpin RNA, IFNγ = interferon gamma, * = *p* < 0.05, ** = *p* < 0.01, *** = *p* < 0.001)

Exposure of PC cell lines to 25 ng/ml IFNγ for 48 h showed a significant increase of PD-L1 expression in all three control and nc- 22Rv1, DU145, and PC3 cell lines (22Rv1 3.27-fold, *p* < 0.001; nc-22Rv1 2.84-fold, *p* < 0.01; DU145 2.8-fold, *p* < 0.001; ncDU145 2.38-fold *p* < 0.001; PC3 2.02-fold, *p* < 0.01; ncPC3 twofold, p < 0.01). The same pattern was observed in shHIF1α 22Rv1, DU145, and PC3 (2.36-fold increase, *p* < 0.001; shDU145 2.89-fold increase, *p* < 0.001; shPC3 2.86-fold increase, *p* < 0.001) (Fig. 4A,B).

The above observation was also confirmed in RT-PCR, where PD-L1 mRNA expression increased in all three 22Rv1, DU145, and PC3 cell lines, control, nc- and shHIF1α ones (22Rv1 2.9-fold increase, *p* < 0.001; nc22Rv1 2.6-fold increase, *p* < 0.01; sh22Rv1 2.19-fold increase *p* < 0.001; DU145 3.5-fold increase, *p* < 0.001; ncDU145 2.7-fold increase, *p* < 0.001,; shDU145 2.8-fold increase, *p* < 0.001; PC3 twofold increase, *p* < 0.01; ncPC3 2.7-fold, *p* < 0.001; shPC3 2.5-fold increase, *p* < 0.001) (Fig. 4C).

## Discussion

The PD-L1/PD-1 immune checkpoint inhibitory pathway is the best studied and targeted for the development of immunotherapy. Monoclonal antibodies (MoAbs) targeting PD-L1 or PD-1 have revolutionized the treatment of advanced cancer [17]. Unlike other solid tumors, anti-PD-L1/PD-1 immunotherapy is still investigational for PC and is mainly approved for mismatch-deficient tumors refractory to hormonal therapy and chemotherapy [18]. The addition of anti-PD-L1/PD-1 immunotherapy to hormonal therapy or chemotherapy has not provided clear evidence of benefit [19, 20]. Although studies with RT combination with anti-PD-L1/PD-1 MoAbs are not available, radiotherapy increased by 2–3-fold the overall survival rates of patients with metastatic PC undergoing ipilimumab (anti-CTLA4 MoAb) immunotherapy [21]. The radio-vaccination effect during RT may significantly enhance the results of immunotherapy.

The identification of subgroups of PC patients expected to benefit from anti-PD-L1/PD-1 immunotherapy would facilitate the conduct of clinical trials in patient subpopulations. Although extensively studied in other malignancies, PD-L1 expression is less studied in PC. It has been reported that PD-L1 is expressed by PC cells and stroma infiltrating immune cells in 10–50% of tumors, pending upon histological subtype and antibodies used for the immunohistochemical detection of the protein [22]. In our study, PD-L1 was expressed by cancer cells in 25% of PCs, while PD-L1 expression by tumor-infiltrating lymphocytes and macrophages was noted in 30% of PCs. As PD-L1 is an inducible gene [23], studying the microenvironmental conditions that are linked with PD-L1 expression is essential to identify subgroups of tumors with distinct biology that drives the PD-L1 expression status.

In the current study, we investigated the hypoxia and anaerobic metabolism/acidity-related gene expression, namely HIF1α and LDHA. A strong direct association of PD-L1 expression by cancer cells with these tumor markers was noted. Cell line experiments that followed confirmed that PD-L1 expression is strongly induced by hypoxia, although not by acidity, even in the hormone-dependent cell line 22Rv1 which had very low steady-state levels of PD-L1. Hypoxia stimulated PD-L1 expression at both transcription and translation levels. This is in accordance with a previously reported study by Xu et al [24]. Similar results have been reported in other types of tumors, like bladder cancer and glioblastoma [25, 26].

An important finding was also the association of HIF1α with low infiltration of PCs with TILs in accordance with previous studies in breast and head–neck cancer [27, 28]. A hypoxic and acidic TME provides a functional barrier against cytotoxic lymphocytes, blocking their proliferation and cytotoxic activity, and furthermore, promotes regulatory T-cell and monocyte prevalence [6–10]. It seems that hypoxia has multiple immunosuppressive effects in the TME, by enhancing PD-L1 expression by cancer cells and repressing cytotoxic T-cell density and activity.

As HIF1α a key transcription factor involved in the adaptation of normal and cancer cells to hypoxia [29], we investigated the hypothesis that hypoxic PD-L1 regulation is HIF dependent. We created three PC cell lines with stably silenced HIF1α gene. Unlike the parental cells, these cells did not up-regulate PD-L1 mRNA and protein under hypoxic conditions, suggesting that PD-L1 is a HIF1α inducible gene. In fact, HIF-silenced cells showed a reduction of the PD-L1 expression, which may suggest that constitutively expressed levels of HIF1α in cancer cells drive PD-L1 expression that can be suppressed by HIF-blockage. In 2014, Noman et al. showed the direct binding of HIF1α (but not ΗΙF2α) to a hypoxia-response element in the promoter of the PD-L1 gene [11]. Targeting HIF1α suppresses PD-L1 expression in experimental studies [30, 31]. For this purpose, we exposed two PC cell lines with high PD-L1 protein and mRNA levels, namely DU145 and PC3, on 50 μmol/L of chrysin, a well-known inhibitor of HIF1α stability [32]. Exposure of the cell lines to chrysin resulted in down-regulation of both HIF1α and PD-L1, in both cell lines indicating the direct relation of a hypoxia-induced pathway on PD-L1 activity in PC. Similar studies [32] reported the down-regulation of PD-L1 via chrysin through the STAT3 (Signal transducer and activator of transcription factor 3) and NF-*κ*B (Nuclear factor kappa B) pathways in hepatocellular carcinoma.

In a subsequent step, we examined the role of the eventual immunogenic radiation-induced death and lymphocytic interactions in the release of soluble factors that could induce PD-L1 expression in viable cancer cells. Direct irradiation and culturing cancer cells with medium from irradiated cancer cells did not up-regulate PD-L1. On the contrary, culturing the cancer cell lines with medium from untreated PBMC cultures from a healthy donor resulted in PD-L1 up-regulation in all cell lines, including the ones with silenced HIF1α gene. It was, therefore, postulated that soluble factors released by lymphocytes induce PD-L1 independently of hypoxia and the HIF1α pathway. As IFNγ has been recognized to potently induce PD-L1 expression [33], we further tested whether this hypoxia-independent pathway is triggered by this cytokine. IFNγ strongly induced the expression of PD-L1 in all three PC cell lines, including the ones with silenced HIF1α gene. The up-regulation of PD-L1 by soluble factors released by lymphocytes should be sought in the activity of other cytokines and chemokines [34–36]. Although RT did not directly up-regulate PD-L1, post-irradiation inflammatory response may induce PD-L1 in PC patients treated with radiotherapy.

**Fig. 5:**
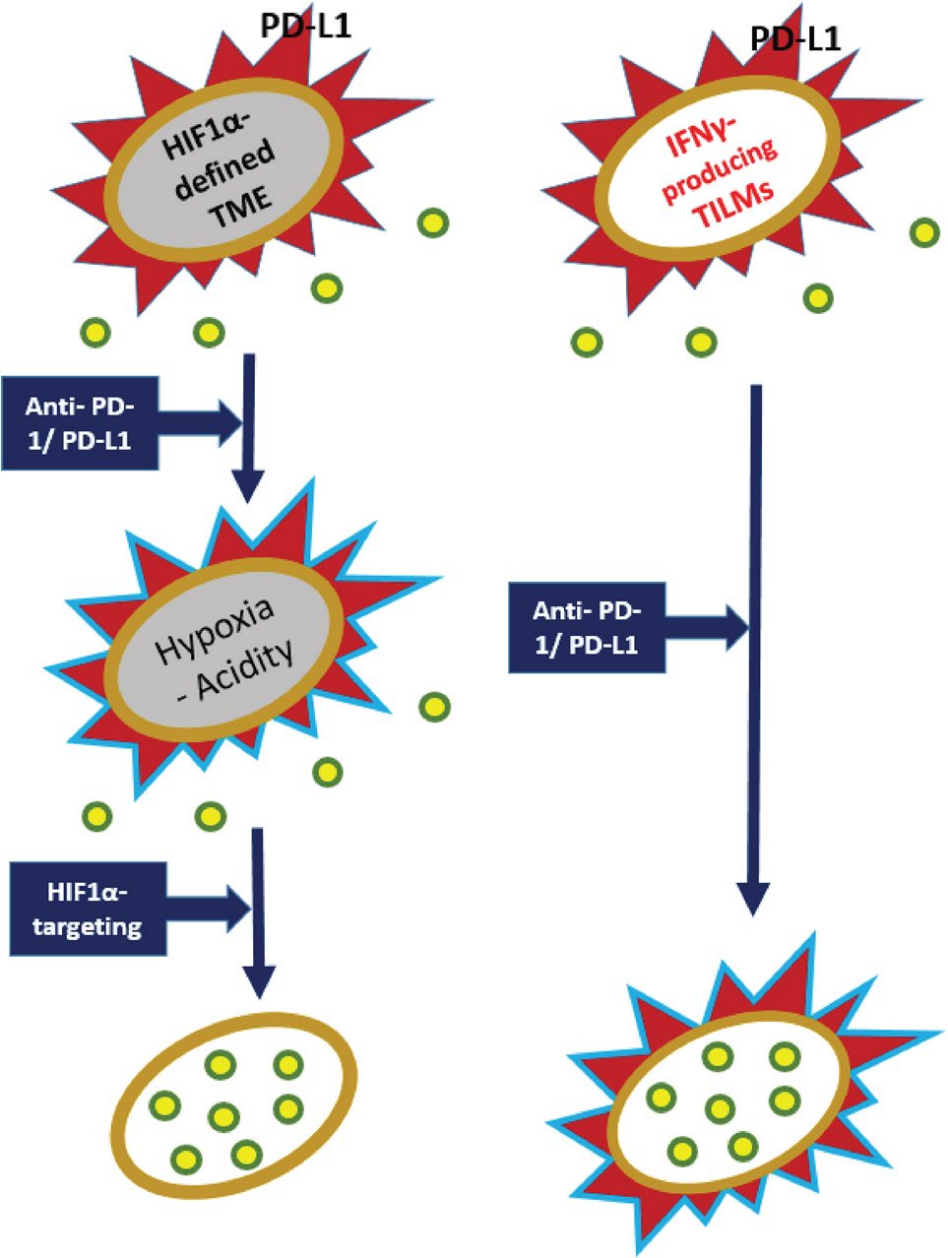
Two distinct cellular pathways regulating PD-L1 up-regulation. *Pathway 1:* HIF1α overexpression, whether hypoxia induced or inherently up-regulated, triggers PD-L1 overexpression on cancer cell membranes, producing a PD-L1-rich tumor (red structure), characterized by a hypoxic and/or acidic TME (gray). Anti-PD-1/PD-L1 immunotherapy neutralizes the anti-immune PD-L1 enrichment (light blue). However, due to hypoxic/acidic conditions, cytotoxic T-cells (yellow/green structures) cannot proliferate and act in this adverse TME. Blockage of HIF1α activity will have a dual effect by repressing PD-L1 expression and reducing anaerobic metabolism and acidity. This may assist cytotoxic T-cells in thriving in the tumor and exerting antitumor cytotoxicity. *Pathway 2:* Constitutive or Radiotherapy-induced IFNγ produced by TILMs up-regulates PD-L1 expression by cancer cells, blocking cytotoxic T-cell activity. Anti-PD-1/PD-L1 immunotherapy blocks PD-L1 and facilitates cytotoxic T-cell activity, provided that this pathway does not co-exist with the hypoxia-driven one

It is concluded that hypoxia induces PD-L1 expression in PC cell lines through a HIF1α-dependent mechanism. Soluble factors released by PBMCs and IFNγ can strongly induce this phenotype regardless of the activity of HIF1α, which may be intensified during radiotherapy of PC patients. We suggest that PD-L1 expression is induced at least by two distinct molecular pathways (Fig. 5). The first is dependent on the hypoxic tumor microenvironment, while the second is cytokine related and eventually driven by the tumor-infiltrating lymphocytes or monocytes in the tumor microenvironment. This finding demands further investigation to assess the cellular components of PBMCs that trigger PD-L1 expression. It is worrying that an intense infiltration of the tumor by TILs, a desirable antitumor immune response, can, at the same time, be the cause of the induction of a cancer defensive mechanism through PD-L1 up-regulation. Hypoxic tumors, also linked with poor TIL density, can escape anti-PD-1/PD-L1 immunotherapy as cytotoxic T-cell proliferation and function are blocked in such TME. Targeting the hypoxic conditions and HIF1α function in combination with anti-PD-L1 immunotherapy can overpass the adverse effect PD-L1, whether attributed to HIF1α or IFNγ in the TME, by enhancing the recognition of cancer cells and allowing effective cancer cell killing by the immune system.

## Data Availability

The datasets generated and analyzed during the current study are available from the corresponding author on reasonable request.

## References

[1] Bordon Y (2015). Tumour immunology: Checkpoint parley. Nat Rev Immunol.

[2] Riella LV, Paterson AM, Sharpe AH, Chandraker A (2012). Role of the PD-1 pathway in the immune response. Am J Transplant.

[3] Ingles Garces AH, Au L, Mason R, Thomas J, Larkin J (2019). Building on the anti-PD1/PD-L1 backbone: combination immunotherapy for cancer. Expert Opin Investig Drugs.

[4] Gatenby RA, Gillies RJ (2004). Why do cancers have high aerobic glycolysis?. Nat Rev Cancer.

[5] Koukourakis MI, Giatromanolaki A (2019). Warburg effect, lactate dehydrogenase, and radio/chemo-therapy efficacy. Int J Radiat Biol.

[6] Huber V, Camisaschi C, Berzi A, Ferro S, Lugini L, Triulzi T, Tuccitto A, Tagliabue E, Castelli C, Rivoltini L (2017). Cancer acidity: An ultimate frontier of tumor immune escape and a novel target of immunomodulation. Semin Cancer Biol.

[7] Labiano S, Palazon A, Melero I (2015). Immune response regulation in the tumor microenvironment by hypoxia. Semin Oncol.

[8] Pilon-Thomas S, Kodumudi KN, El-Kenawi AE, Russell S, Weber AM, Luddy K, Damaghi M, Wojtkowiak JW, Mulé JJ, Ibrahim-Hashim A, Gillies RJ (2016). Neutralization of Tumor Acidity Improves Antitumor Responses to Immunotherapy. Cancer Res.

[9] Bosticardo M, Ariotti S, Losana G, Bernabei P, Forni G, Novelli F (2001). Biased activation of human T lymphocytes due to low extracellular pH is antagonized by B7/CD28 costimulation. Eur J Immunol.

[10] Lunt SY, Vander Heiden MG (2011). Aerobic glycolysis: meeting the metabolic requirements of cell proliferation. Annu Rev Cell Dev Biol.

[11] Noman MZ, Desantis G, Janji B, Hasmim M, Karray S, Dessen P, Bronte V, Chouaib S (2014). PD-L1 is a novel direct target of HIF-1α, and its blockade under hypoxia enhanced MDSC-mediated T cell activation. J Exp Med.

[12] Jiang X, Wang J, Deng X, Xiong F, Ge J, Xiang B, Wu X, Ma J, Zhou M, Li X, Li Y, Li G, Xiong W, Guo C, Zeng Z (2019). Role of the tumor microenvironment in PD-L1/PD-1-mediated tumor immune escape. Mol Cancer.

[13] Chen J, Feng Y, Lu L, Wang H, Dai L, Li Y, Zhang P (2012). Interferon-γ-induced PD-L1 surface expression on human oral squamous carcinoma via PKD2 signal pathway. Immunobiology.

[14] Imai Y, Chiba T, Kondo T, Kanzaki H, Kanayama K, Ao J, Kojima R, Kusakabe Y, Nakamura M, Saito T, Nakagawa R, Suzuki E, Nakamoto S, Muroyama R, Tawada A, Matsumura T, Nakagawa T, Kato J, Kotani A, Matsubara H, Kato N (2020). Interferon-γ induced PD-L1 expression and soluble PD-L1 production in gastric cancer. Oncol Lett.

[15] Koukourakis MI, Giatromanolaki A, Panteliadou M, Pouliliou SE, Chondrou PS, Mavropoulou S, Sivridis E (2014). Lactate dehydrogenase 5 isoenzyme overexpression defines resistance of prostate cancer to radiotherapy. Br J Cancer.

[16] Koukourakis MI, Kakouratos C, Kalamida D, Bampali Z, Mavropoulou S, Sivridis E, Giatromanolaki A (2016). Hypoxia-inducible proteins HIF1α and lactate dehydrogenase LDH5, key markers of anaerobic metabolism, relate with stem cell markers and poor post-radiotherapy outcome in bladder cancer. Int J Radiat Biol.

[17] Gong J, Chehrazi-Raffle A, Reddi S, Salgia R (2018). Development of PD-1 and PD-L1 inhibitors as a form of cancer immunotherapy: a comprehensive review of registration trials and future considerations. J Immunother Cancer.

[18] Antonarakis ES, Piulats JM, Gross-Goupil M, Goh J, Ojamaa K, Hoimes CJ, Vaishampayan U, Berger R, Sezer A, Alanko T, de Wit R, Li C, Omlin A, Procopio G, Fukasawa S, Tabata KI, Park SH, Feyerabend S, Drake CG, Wu H, Qiu P, Kim J, Poehlein C, de Bono JS (2020). Pembrolizumab for treatment-refractory metastatic castration-resistant prostate cancer: multicohort, open-label phase II KEYNOTE-199 study. J Clin Oncol.

[19] Fizazi K, González Mella P, Castellano D, Minatta JN, Rezazadeh Kalebasty A, Shaffer D, Vázquez Limón JC, Sánchez López HM, Armstrong AJ, Horvath L, Bastos DA, Amin NP, Li J, Unsal-Kacmaz K, Retz M, Saad F, Petrylak DP, Pachynski RK (2022). Nivolumab plus docetaxel in patients with chemotherapy-naïve metastatic castration-resistant prostate cancer: results from the phase II CheckMate 9KD trial. Eur J Cancer.

[20] Powles T, Yuen KC, Gillessen S, Kadel EE, Rathkopf D, Matsubara N, Drake CG, Fizazi K, Piulats JM, Wysocki PJ, Buchschacher GL, Alekseev B, Mellado B, Karaszewska B, Doss JF, Rasuo G, Datye A, Mariathasan S, Williams P, Sweeney CJ (2022). Atezolizumab with enzalutamide versus enzalutamide alone in metastatic castration-resistant prostate cancer: a randomized phase 3 trial. Nat Med.

[21] Fizazi K, Drake CG, Beer TM, Kwon ED, Scher HI, Gerritsen WR, Bossi A, den Eertwegh AJMV, Krainer M, Houede N, Santos R, Mahammedi H, Ng S, Danielli R, Franke FA, Sundar S, Agarwal N, Bergman AM, Ciuleanu TE, Korbenfeld E, Sengeløv L, Hansen S, McHenry MB, Chen A, Logothetis C (2020). CA184–043, Investigators Final Analysis of the Ipilimumab Versus Placebo Following Radiotherapy Phase III Trial in Postdocetaxel Metastatic Castration-resistant Prostate Cancer Identifies an Excess of Long-term Survivors. Eur Urol.

[22] Palicelli A, Bonacini M, Croci S, Magi-Galluzzi C, Cañete-Portillo S, Chaux A, Bisagni A, Zanetti E, De Biase D, Melli B, Sanguedolce F, Ragazzi M, Bonasoni MP, Soriano A, Ascani S, Zizzo M, Castro Ruiz C, De Leo A, Giordano G, Landriscina M, Carrieri G, Cormio L, Berney DM, Athanazio D, Gandhi J, Cavazza A, Santandrea G, Tafuni A, Zanelli M (2021). What do we have to know about PD-l1 expression in prostate cancer? A systematic literature review. Part 1: focus on immunohistochemical results with discussion of pre-analytical and interpretation variables. Cells.

[23] Sun C, Mezzadra R, Schumacher TN (2018). Regulation and Function of the PD-L1 Checkpoint. Immunity.

[24] Xu LJ, Ma Q, Zhu J, Li J, Xue BX, Gao J, Sun CY, Zang YC, Zhou YB, Yang DR, Shan YX (2018). Combined inhibition of JAK1,2/Stat3-PD-L1 signaling pathway suppresses the immune escape of castration-resistant prostate cancer to NK cells in hypoxia. Mol Med Rep.

[25] Ding XC, Wang LL, Zhang XD, Xu JL, Li PF, Liang H, Zhang XB, Xie L, Zhou ZH, Yang J, Weichselbaum RR, Yu JM, Hu M (2021). The relationship between expression of PD-L1 and HIF-1α in glioma cells under hypoxia. J Hematol Oncol.

[26] Smith V, Mukherjee D, Lunj S, Choudhury A, Hoskin P, West C, Illidge T (2021). The effect of hypoxia on PD-L1 expression in bladder cancer. BMC Cancer.

[27] Giatromanolaki A, Gkegka AG, Pouliliou S, Biziota E, Kakolyris S, Koukourakis M (2022). Hypoxia and anaerobic metabolism relate with immunologically cold breast cancer and poor prognosis. Breast Cancer Res Treat.

[28] Koukourakis IM, Gkegka AG, Xanthopoulou E, Nanos C, Giatromanolaki A, Koukourakis MI (2022). Prognostic and predictive relevance of tumor-infiltrating lymphocytes in squamous cell head-neck cancer patients treated with radical radiotherapy/chemo-radiotherapy. Curr Oncol.

[29] Semenza GL (2003). Targeting HIF-1 for cancer therapy. Nat Rev Cancer.

[30] Bailey CM, Liu Y, Liu M, Du X, Devenport M, Zheng P, Liu Y, Wang Y (2022). Targeting HIF-1α abrogates PD-L1-mediated immune evasion in tumor microenvironment but promotes tolerance in normal tissues. J Clin Invest.

[31] Zhao Y, Wang XX, Wu W, Long H, Huang J, Wang Z, Li T, Tang S, Zhu B, Chen D (2019). EZH2 regulates PD-L1 expression via HIF-1α in non-small cell lung cancer cells. Biochem Biophys Res Commun.

[32] Fu B, Xue J, Li Z, Shi X, Jiang BH, Fang J (2007). Chrysin inhibits expression of hypoxia-inducible factor-1alpha through reducing hypoxia-inducible factor-1alpha stability and inhibiting its protein synthesis. Mol Cancer Ther.

[33] Mandai M, Hamanishi J, Abiko K, Matsumura N, Baba T, Konishi I (2016). Dual faces of IFNγ in cancer progression: a role of PD-L1 Induction in the determination of pro- and antitumor immunity. Clin Cancer Res.

[34] Xu L, Chen X, Shen M, Yang DR, Fang L, Weng G, Tsai Y, Keng PC, Chen Y, Lee SO (2018). Inhibition of IL-6-JAK/Stat3 signaling in castration-resistant prostate cancer cells enhances the NK cell-mediated cytotoxicity via alteration of PD-L1/NKG2D ligand levels. Mol Oncol.

[35] Wang X, Yang L, Huang F, Zhang Q, Liu S, Ma L, You Z (2017). Inflammatory cytokines IL-17 and TNF-α up-regulate PD-L1 expression in human prostate and colon cancer cells. Immunol Lett.

[36] Carbotti G, Barisione G, Airoldi I, Mezzanzanica D, Bagnoli M, Ferrero S, Petretto A, Fabbi M, Ferrini S (2015). IL-27 induces the expression of IDO and PD-L1 in human cancer cells. Oncotarget.

